# Targeted Removal of Ant Colonies in Ecological Experiments, Using Hot Water

**DOI:** 10.1673/031.007.4101

**Published:** 2007-07-09

**Authors:** Walter R. Tschinkel, Joshua R. King

**Affiliations:** Department of Biological Science, Florida State University, Tallahassee, FL 32306-4370

**Keywords:** fire ant, *Solenopsis invicta*

## Abstract

Ecological experiments on fire ants cannot, or should not, use poison baits to eliminate the fire ants because such baits are not specific to fire ants, or even to ants. Hot water is an extremely effective and specific killing agent for fire ant colonies, but producing large amounts of hot water in the field, and making the production apparatus mobile have been problematical. The construction and use of a charcoal-fired kiln made from a 55-gal. oil drum lined with a sand-fireclay mixture is described. An automobile heater fan powered from a 12-v battery provided a draft. Dual bilge pumps pumped water from a large tank through a long coil of copper tubing within the kiln to produce 4 to 5 l. of hot water per min. The hot water was collected in 20 l. buckets and poured into fire ant nests previously opened by piercing with a stick. The entire assembly was transported in and operated from the back of a pickup truck.

Five experimental plots containing 32 to 38 colonies of the fire ant, *Solenopsis invicta*, Buren (Hymenoptera: Formicidae), were treated with hot water over a period of two years. All colonies on the treatment plots were treated twice with hot water early in 2004, reducing their numbers to zero. However new colonies were formed, and mature colonies expanded into the plots. A third treatment was made in the spring of 2005, after which fire ant populations were suppressed for over a year. Whereas the 5 control plots contained a total of 166 mostly large colonies, the 5 treatment plots contained no live colonies at all. Averaged over a two-year period, a 70% reduction in total number of colonies was achieved (*P* < 0.001) on the treatment plots, and a 93% reduction of large, mature colonies. Over this same time span, the number of colonies in control plots remained stable. The reduction in colony numbers on the treatment plots was reflected in the pitfall trap samples that recorded a 60% reduction in fire ants.

## Introduction

**Figure 1A.**
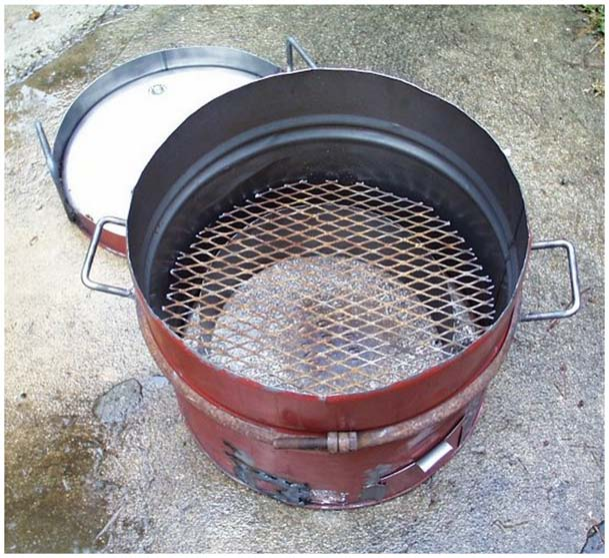
Oil drum and lid, before lining with fire clay.

Experiments that test the ecological effects of an organism are best carried out by removing that organism from the experimental plots, and then comparing the removal with intact control plots. The experimental removal of arthropods is notoriously difficult and often relies on the application of broad-spectrum insecticides (e.g., Wilson and Simberloff 1969). Many social insects, in spite of being relatively easily located and manipulated due to their colonial existence, have also been most commonly manipulated in the field using only insecticides (e.g., Ryti and Case 1988). Experiments on the fire ant, *Solenopsis invicta* Buren (Hymenoptera: Formicidae), one of the most studied social insects, have almost universally relied on poison baits to remove or reduce populations of this ant. A few examples will suffice: Seagraves et al. (2004) killed fire ants with three different bait treatments to test their effect on arthropod ground predators; Allen (1995) reduced fire ant populations with Amdro® bait to determine their effect on bobwhite quail; and Reagan et al. (1972) reduced fire ants with Mirex® bait to determine their effect on sugar cane pests.

None of these poison baits are specific to fire ants as they kill other species of ants indiscriminately, and often kill scavenging insects such as crickets and many other invertebrates (Markin et al. 1974a; Markin et al. 1974b; Reagan et al. 1972; Wojcik et al. 1975). Moreover, secondary intoxication from eating poisoned ants or insects may occur. Ascribing any observed effects to the removal of fire ants is therefore unsound. When the intended response variable is the species richness or abundance of the native ant community, the use of poison baits to remove fire ants is obviously even more problematical. As a result, there have been no experiments testing the effects of *S. invicta* on native ants, even though it is widely believed that *S. invicta* suppresses native ant populations.

Wishing to test the effects of *S. invicta* on native ant populations, we needed a method for killings. *invicta* that did not rely on poison baits, and did not kill co-occurring ants. Because insects are small, they heat quickly, ensuring that even brief contact with high temperatures are often lethal. Furthermore, the structure of fire ant nests consists of multiple interconnected shafts and chambers in which the queen, the brood and majority of workers reside in close proximity, making it possible to kill entire colonies with hot water. Tschinkel and Howard (1980) demonstrated that hot water poured into nests killed colonies effectively, and Adams and Tschinkel (2001) used this method in an experiment on fire ant population regulation. In both studies, water was heated at a fixed location in a large pot, limiting the number and spatial distribution of treated colonies. In these experiments on fire ants and native ants, treatment plots were widely scattered and contained many hundreds of colonies, making a fixed location, low volume, batch-wise heating system impractical. To meet this challenge, a flow-through charcoal kiln was developed that produces a continuous flow of hot water, limited only by the size of the reserve tank. Here, we report its construction and use over two years to suppress populations of *S. invicta*.

**Figure 1B.**
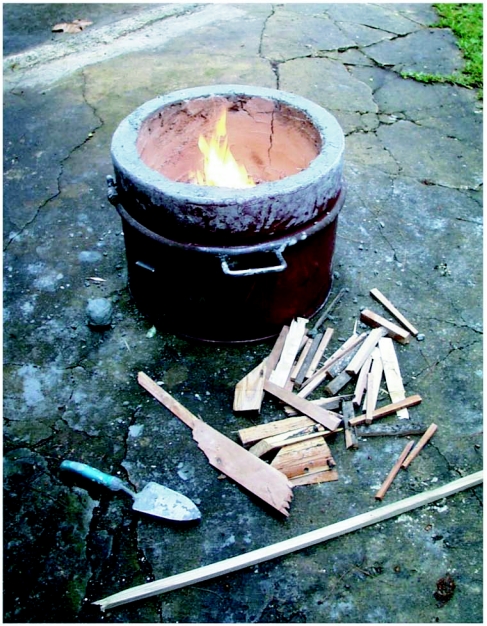
The lined kiln is being dried with a small fire.

## Materials and Methods

### Kiln construction

**Figure 2.**
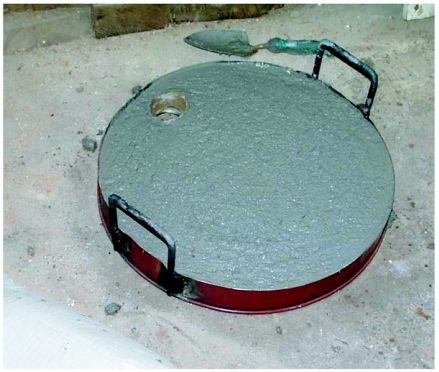
The kiln lid filled with sand/fireclay mixture, before drying and firing.

The kiln was constructed of the bottom half of a 55-gal. steel oil drum, lined with a 6 to 7 cm thick layer of a 2:1 sand/fire clay mixture (Figure 1A, 1B). The bottom 15 cm. of the wall was not lined with fireclay, but was provided with a metal baffle that lined up with the inner surface of the kiln lining. This baffle both saved weight and supported a grid made of expanded steel on which the charcoal fuel rested (Figure 1A). Near the bottom, a hole admitted a blast of air from a converted automobile heater-fan that was powered by a 12-v deep-discharge battery (marine battery). A door next to the air vent allowed ash cleanout. A lid was fashioned from the top of the oil drum, cut so that a 5 cm rim remained, that was filled with a sand/fire clay mixture for insulation (Figure 2). The filling-bung formed the chimney vent.

**Figures 3.**
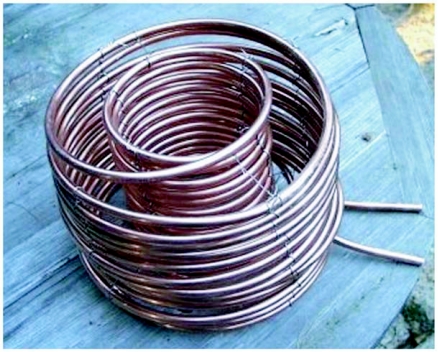
Flow-through copper tubing coil for heating water in the kiln. The loops of the coil are tied together with stainless steel wire.

The wet clay lining was dried with a moderate charcoal fire until it stopped steaming. The heat from the fire was then raised to a maximum by increasing the fuel load and airflow. This partially vitrified the walls.

### Heating coil

**Figure 4.**
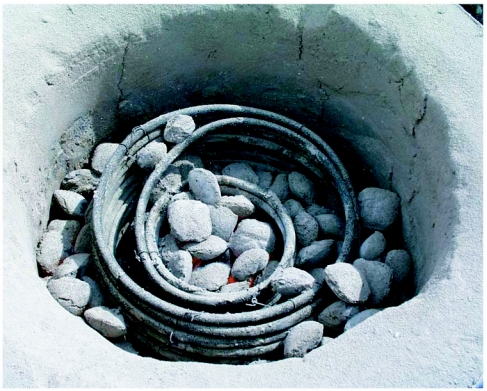
The coil in operation in the kiln, lid removed. Note that charcoal contacts both the inner and outer sides of both coils.

The water-heating coil was made by wrapping 18 m of 3/8 inch outer diameter soft copper tubing around cylinders and tying the stacked coils together with stainless steel wire (Figure 3). A 28 cm diameter outer coil surrounded a 15 cm inner coil, leaving enough space for charcoal briquettes to fit between them (Figure 4). The open ends of the tubing projected through a port in the wall of the kiln. One was connected to the water inflow, and other to the outflow.

**Figure 5.**
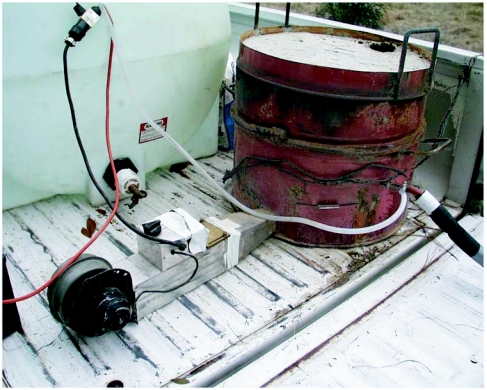
Modified automobile heater fan, powered by a 12-v marine battery. The tapered outlet increases the air velocity entering the bottom of the kiln.

**Figure 6.**
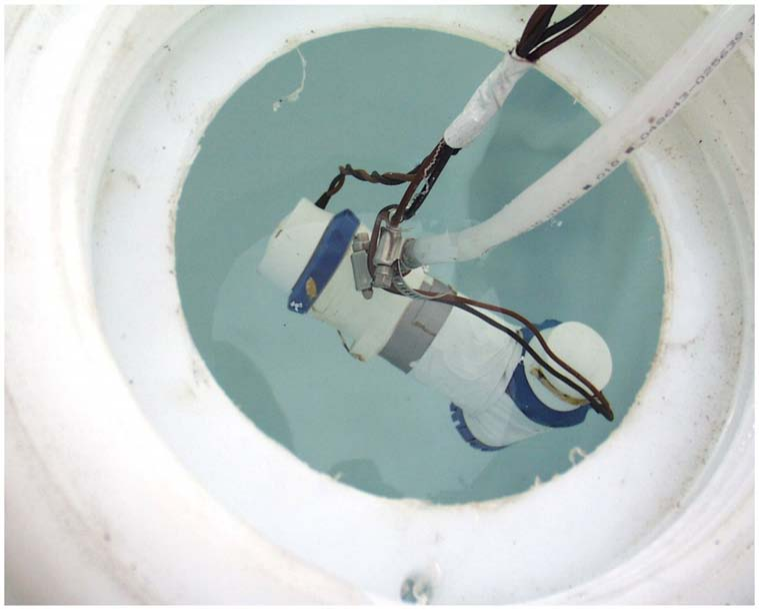
Dual, in-series bilge pumps inside the 225-gallon water tank.

### Air supply

A 12-v squirrel cage fan from an automobile heater system supplied the airflow. The fan was converted to an air blower using an aluminum sheet metal case and a variable speed switch (Figure 5). The fan output end was tapered to increase the air speed before entering the kiln through the vent-hole below the charcoal and flowing upward through the charcoal. Varying the speed of the fan with a 3-stage switch permitted regulation of the burn-rate within the kiln.

### Water supply

Water was pumped from a 225-gallon plastic tank (Figure 6) by means of two 1500 gallon-per-hr bilge pumps in series, powered by the 12-v battery that was also used for the blower. The bilge pumps were connected to the inflow tube of the kiln by plastic tubing with 3/8^th^ in. inner diameter. In spite of this apparently excess pumping capacity, the resistance of 18 m of tubing limited output flow rate to about 4–5 liters per min.

### Ash removal

The binding material used to make charcoal briquettes leaves behind a great deal of ash. This had to be periodically shaken down and raked out through the ash-removal door at the base of the kiln.

### Kiln Operation

**Figure 7.**
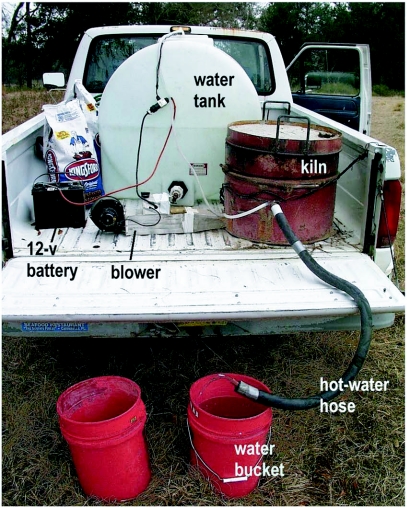
The entire assembly in operation in a pickup truck. Hot water flows continuously from the insulated hose exiting from the kiln. It is collected in the bucket and then dispensed onto a fire ant nest.

**Figure 8.**
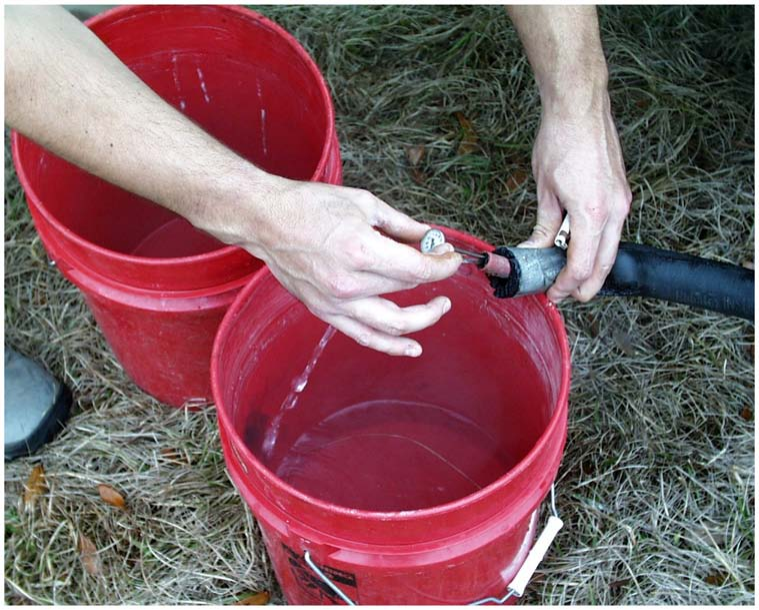
Measuring the temperature at the hot-water outlet. Knocking down ash and keeping the charcoal topped up assures that the effluent water temperature is always between 60° and 85° C.

The tank, battery, fan, charcoal supply and kiln were loaded onto the back of a pickup truck and driven to the field plots (Figure 7). The charcoal was ignited in the kiln with kindling, and the water flow started as soon as the coil warmed. Because the water was pumped at low-pressure, this early water-flow was necessary to prevent a steam-lock and excessive oxidation of the copper. When the system was in full operation, 70 to 85°C water flowed from the outlet at the rate of 4 to 5 liters per minute, filling a 20 liter plastic bucket in 5 minutes or so. The output water temperature was frequently monitored with a thermometer (Figure 8), and the airflow and charcoal fill adjusted to maintain a suitably high temperature. Occasional agitation of the charcoal fill caused the accumulated ash to fall through the grate, increasing heat transfer efficiency and water temperature. The truck could be driven from site to site without interrupting operation of the kiln, but water-flow had to be continuous to prevent steam locks.

**Figure 9.**
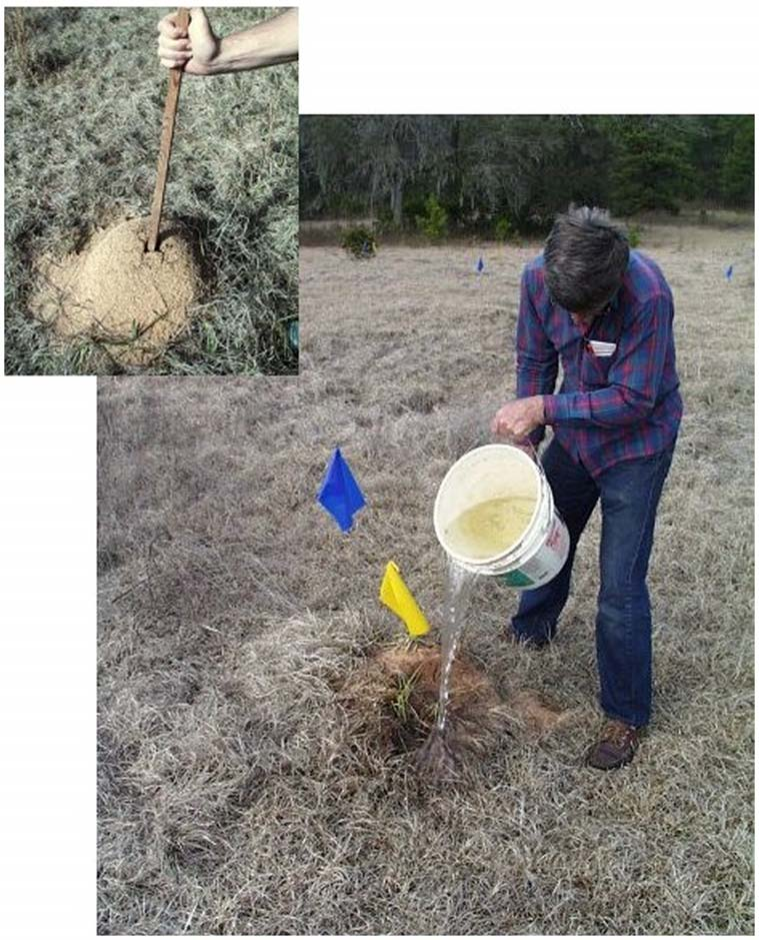
Pouring hot water into a fire ant colony that has been opened by piercing subterranean chambers with a stick.

The full bucket of hot water was carried to the next fire ant nest. The nest was opened by driving a stick downward through the subterranean nest chambers, and the hot water was rapidly poured into the resulting hole until the nest was full (Figure 9). The large number of interconnected chambers and vertical shafts assured that the complete nest was filled quickly with hot water, killing all the ants contacted. The remaining hot water was used to collapse the mound. Very large colonies sometimes required two or even three buckets of water, but small colonies could be killed with a quarter to a half-bucket.

The tank held enough water (850 liters) to treat 40 to 50 colonies, about 3 to 4 hours of work. Heating this water consumed 25 to 30 kg of charcoal.

### Experimental plots

The 10 experimental plots (5 control and 5 experimental) measured 40-by-40 m and were located in pastureland. They contained 32 to 38 colonies each, so that two plots could be treated in a long day's work. Inspection after several weeks revealed many surviving nest fragments, perhaps missed in the nest, but mostly probably reassembled from foragers that were in the field at the time of the killing. Such surviving fragments were killed with a second treatment.

### Assessment of ant populations

Fire ant colony populations were assessed by direct counts of active mounds. Colonies were classified as either small or large by visually estimating mound volume. Large colonies were those that exceeded approximately 5–7 liters in volume, corresponding to a colony of approximately 50, 000 to 70, 000 workers (Tschinkel 2006). During early August of both years, an array of 36 pitfalls was placed in each plot, arranged in a 6 × 6 grid with 5 m between traps. No trap was closer than 7.5 m from any boundary. The pitfalls were straight-sided plastic tubes with inside dimensions of 30 mm diam. × 85 mm. Propylene glycol was added to each trap to a height of about 15 mm, the traps inserted flush with the ground surface and left open for one week. The catch in these pitfalls estimated the activity of both fire ants and other co-occurring species. Data were analyzed by analysis of variance, and transformed as necessary to stabilize the variance.

## Results

The kill rate was excellent, and the fire ant population was reduced by at least as much as reported in studies using poison baits (e.g., Cook, 2003). Unlike poison baits (and to a lesser degree, mound treatment with poisons), the use of hot water specifically reduced fire ants, and only fire ants (data reported in King and Tschinkel, 2006). At the beginning of the study in 2004, there were 155 colonies on the 5 treatment plots combined (average per plot 31; range: 25 to 40). Of these, 105 were large, mature colonies (average per plot 21; range: 14 to 26). All colonies on the treatment plots were treated twice with hot water early in 2004, reducing their numbers to zero.

**Figure 10.**
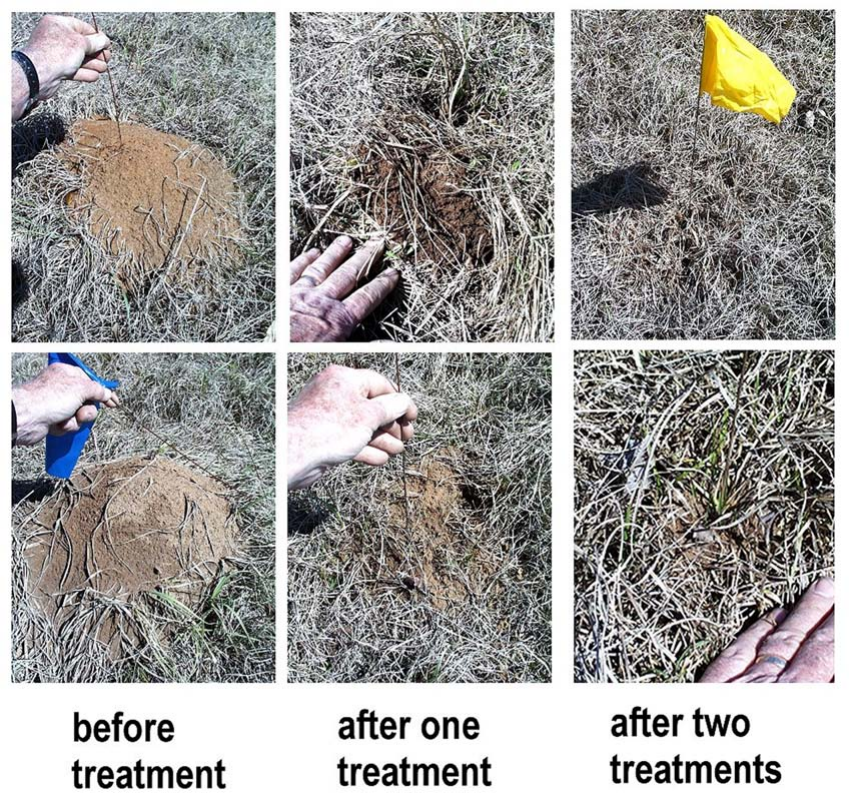
Typical aspects of colonies before treatment with hot water, and after one and two treatments. Most colonies were queenless after one treatment, and had only a few surviving workers, if any, after two treatments.

**Figure 11.**
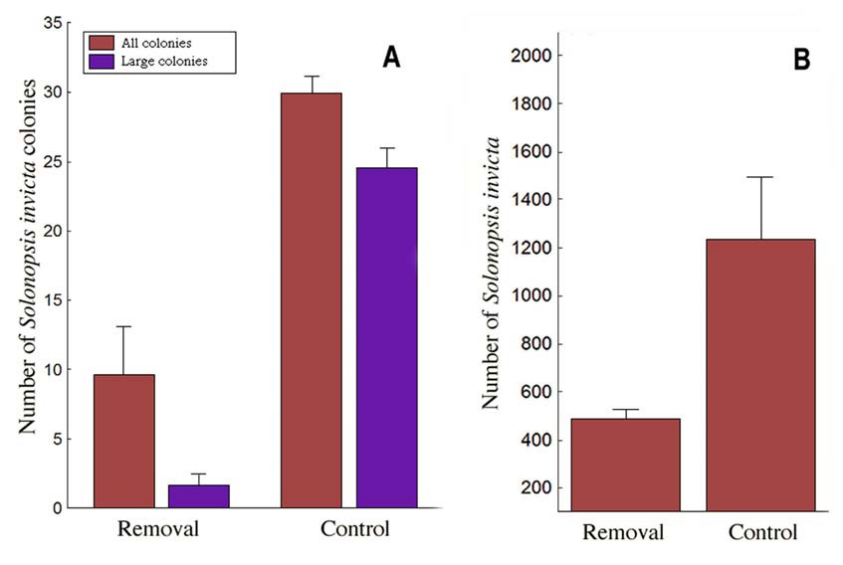
A. The mean number of colonies, especially large colonies, was greatly reduced on treatment plots. On control plots, most colonies are large and mature. B. Capture rate of *S. invicta* per plot was much lower on treated plots even 7 months after the first treatments in both years. Data are means for both years. Error bars = 1 S.E.

However, this situation is not stable because new colonies are founded during the spring and summer (April-July), their mounds becoming visible in the following winter/spring (Nov.-March), and mature colonies move in from the perimeter, especially in late winter and early spring (Dec.-Feb.). Thus, in January 2005, 10 months after the first year's treatment, all 5 treatment plots together contained a total of 154 colonies, but only 10% (15 colonies; average per plot 3) of these were mature, that is, large to very large. These mature colonies were probably move-ins. All colonies on treatment plots were then treated twice with hot water. Two weeks later 54 (35%) of the 154 colonies were dead, 85 (55%) has some workers but no worker brood, indicating they were queenless, and 13 (8%) had both workers and worker brood, indicating queenright status. All of these colonies were small (Figure 10), and were eliminated with a third hot water treatment. Thereafter, whereas the 5 control plots contained a total of 166 mostly large colonies, the 5 treatment plots contained no live colonies at all. Averaged over the two-year period, a 70% reduction in total number of colonies [treatment, *F* (1, 23) = 19.37, *P* < 0.001] occurred on the treatment plots, and a 93% reduction of large, mature colonies (Figure 11A). Over this same time span, the number of colonies in control plots remained stable.

The reduction in colony numbers on the treatment plots was reflected in the pitfall trap samples (Figure 11B). Five to seven months after treatment in both years, the capture rate of foraging fire ants in pitfall traps on treated plots averaged 16/pitfall, and on untreated plots 41.5 ± 3.3/pitfall, a 60% reduction [treatment, *F* (1, 20) = 12.59, *P* = 0.003; year *F* (1, 20) = 4.54, *P* = 0.049]. On treatment plots, groups of adjacent perimeter pitfalls often had higher rates of capture suggesting that many of these ants were foraging into the plots from outside them.

As already noted, the absence of fire ants from pasture is not a stable situation. Repeating the treatments annually eliminated newly founded colonies and colonies that had moved into the plots, keeping the fire ant population greatly reduced for most of each year.

## Discussion

The system reported above was the end product of several less successful prototypes. Initially, we intended to kill colonies by piping superheated steam into the nests by means of an injection wand. Although steam was successfully generated using a small, charcoal-fired kiln, steam was not effective in killing colonies because it condensed within centimeters of the injection site, producing only very local kill. We therefore switched to using hot water to kill the nests. The first successful apparatus used a modified metal-melting, field-portable, charcoal-fired kiln fitted with an 18 m copper tubing coil. However, the outer copper coil contacted the inner wall of the kiln so that the coil was heated only on its inner side. This made it difficult to heat water continuously above about 65 to 70° C. A shorter kiln was then designed with a larger inner diameter so that all surfaces of the coil contacted burning charcoal. This report is about this final version of the kiln.

When it comes to killing fire ant colonies, hot water is clearly less convenient and much more involved than poison baits. However, if the intention of an experiment is to determine the effect of fire ants (or any other ant species) on any community, commodity or creature, the use of poison baits makes it uncertain whether the observed effects resulted from the demise of fire ants, that of any of a number of other insects, especially prey or other ants, or the direct effect of the bait. Investigators have rarely drawn attention to this uncertainty, but it is especially acute when the response variable includes ants, many of which are probably as susceptible to poison bait as are fire ants. In such studies, it is necessary specifically to remove fire ants, and only fire ants. Fortunately, the large mound and subterranean nest make *S. invicta* both conspicuous and an easy target for the hot water method.

When colonies are directly treated with a contact poison (e.g., Gibbons and Simberloff, 2005), the problem is perhaps less acute than with poison baits, but it is not absent. Ants killed by poison are available to scavengers who may in turn be intoxicated, and other creatures can contact the poison directly (Markin et al., 1974a, b). Hot water avoids such problems.

During much of the year, it would have been unnecessary to kill queenless worker fragments with a third hot water treatment. However, during February and March, fire ant colonies emit overwintering sexuals on small mating flights. The newly mated queens seek out orphaned nests in order to parasitize the labor of their workers to found their own colony (Tschinkel 1996). Therefore, during the spring treatments, the queenless fragments were killed to prevent this from happening.

We believe that similar success may be attained using this system to kill other species of ants (and perhaps other insects), provided the architecture of their nests has the right characteristics. Several native ant species in north Florida have these characteristics, including several species of *Formica*, and *Aphaenogaster*, as well as *Dolichoderus mariae*, and *Pogonomyrmex badius*. Obviously, using the system to control arboreal or polydomous species would be much more challenging. However, for many monodomous, ground-dwelling or subterranean species this system affords the mobility and volume to manipulate a great number of colonies over a large area. Most importantly, this method of manipulating ant colonies decreases the variability of removal treatments and is much m ore ecologically responsible.
